# High Definition Colonoscopy Combined with i-SCAN Imaging Technology Is Superior in the Detection of Adenomas and Advanced Lesions Compared to High Definition Colonoscopy Alone

**DOI:** 10.1155/2015/167406

**Published:** 2015-06-18

**Authors:** Erik A. Bowman, Patrick R. Pfau, Arnab Mitra, Mark Reichelderfer, Deepak V. Gopal, Benjamin S. Hall, Mark E. Benson

**Affiliations:** ^1^Department of Medicine, University of Wisconsin Hospital and Clinics, 1685 Highland Avenue, Madison, WI 53705, USA; ^2^Division of Gastroenterology and Hepatology, University of Wisconsin Hospital and Clinics, 1685 Highland Avenue, Madison, WI 53705, USA

## Abstract

*Background*. Improved detection of adenomatous polyps using i-SCAN has mixed results in small studies. Utility of i-SCAN as a primary surveillance modality for colorectal cancer screening during colonoscopy is uncertain. *Aim*. Comparing high definition white light endoscopy (HDWLE) to i-SCAN in their ability to detect adenomas during colonoscopy. *Methods*. Prospective cohort study of 1936 average risk patients who had a screening colonoscopy at an ambulatory procedure center. Patients underwent colonoscopy with high definition white light endoscopy withdrawal versus i-SCAN withdrawal during endoscopic screening exam. Primary outcome measurement was adenoma detection rate for i-SCAN versus high definition white light endoscopy. Secondary measurements included polyp size, pathology, and morphology. *Results*. 1007 patients underwent colonoscopy with i-SCAN and 929 with HDWLE. 618 adenomas were detected in the i-SCAN group compared to 402 in the HDWLE group (*p* < 0.01). More advanced adenomas (≥10 mm) were found by i-SCAN, 79 versus 47 (*p* = 0.021) and based upon histology alone 37 versus 18 (*p* = 0.028). *Conclusions*. i-SCAN detected significantly more adenomas and advanced adenomas compared to high definition white light endoscopy.

## 1. Introduction

Colorectal cancer is a leading cause of cancer related deaths in the US today. It is the second most common cause of cancer mortality in the US and is the third most common cancer among US men and women [1]. Most colorectal cancers are presumed to have arisen from adenomatous polyps [2]. Interruption of the progression from adenoma to carcinoma can be achieved by removal of adenomas. The risk of developing colorectal cancer can be reduced via endoscopic resection of adenomas [3, 4].

Various different endoscopic imaging modalities have been developed to optimize the detection of mucosal abnormalities and adenomatous polyps. Examples of the different technologies include chromoendoscopy, high definition white light endoscopy (HDWLE), Fujinon Intelligent Chromo Endoscopy (FICE), autofluorescence imaging (AFI), and narrow band imaging (NBI) [5].

## 2. Background

One of the novel technologies, i*-*SCAN (Pentax, Tokyo, Japan), is a postprocessing software filter designed to improve tissue contrast with three different algorithm modes to improve image enhancement. The different modes aim to highlight different mucosal irregularities and include surface enhancement (SE), contrast enhancement (CE), and tone enhancement (TE). SE improves light and dark contrast to improve visualization of borders between normal and abnormal mucosa and has been suggested as a method to aid in polyp detection. CE results in a slightly blue tinted image from the suppression of certain red and green wavelengths in the white light spectrum to help augment differences in depth within the mucosal surface. Lastly, TE dampers the dominant red light wavelengths to better define subtle mucosal irregularities and vessel structures [6]. The purpose of this study was to compare high definition colonoscopy with i*-*SCAN mode 1 (SE + CE) to standard high definition colonoscopy in the ability to detect adenomas polyps.

## 3. Methods

Average risk patients, without history of previous polypectomy, who were undergoing screening colonoscopy were included. The colonoscopies were performed by 12 experienced gastroenterologists, without fellows, at a single ambulatory procedure center. Patients were excluded if they had known inflammatory bowel disease or if they had a personal or family history of colon cancer. Study patients underwent screening colonoscopy over a 6-month period using a Pentax HD+ i*-*SCAN system in mode 1 (SE + CE).

All patients drank a 3-4 liter polyethylene glycol electrolyte solution for bowel preparation. Conscious sedation was achieved using midazolam, fentanyl, and occasional diphenhydramine administered by experienced endoscopy nurses. After cecal intubation was achieved the endoscopy nurse activated the i*-*SCAN processor in mode 1. Mode 1 remained activated throughout the withdrawal process. No additional i*-*SCAN modes were allowed during the procedure as the other modes are used to help differentiate between polyp types and not used primarily for screening or detection. The endoscopists involved received training on the use of i-SCAN prior to the study. Patient demographics, procedural and polyp data were recorded including polyp pathology, size (<5, 6–9 and ≥10 mm), location, and morphology. The study data was compared to control data collected at the same ambulatory surgery center in patients undergoing screening colonoscopy using standard high definition imaging only on colonoscope withdrawal over a previous 9-month time frame. The study was approved by the University of Wisconsin Institutional Review Board.

### 3.1. Statistical Analysis

The analysis was focused on comparisons between the cohorts of patients screened by colonoscopy with i*-*SCAN to those screened by colonoscopy with standard high definition imaging. Comparisons were made using Student's *t*-test for continuous outcomes and *χ* ^2^ analysis for categorical outcomes. Statistical significance was considered at a two-sided *p* value < 0.05.

## 4. Results

There were 1,936 patients included in the study, 1007 patients in the i-SCAN cohort, and 929 in the control cohort. The mean age for the HD+ i-SCAN group was 55 ± 6.2 and 49% of the study participants were male. For the standard high definition group mean age was 57 ± 7.5 (*p* < 0.001) and 45% were male (*p* = 0.082). The bowel preparation was good/excellent in 74.8% of the i-SCAN group and 79.3% in the control group (*p* = 0.02, Table 1). Mean withdrawal time for patients without polyps detected was 11.04 minutes in the i-SCAN group and 7.97 in the control group (*p* < 0.001).

Polyps and adenomas were consistently detected more frequently in the i-SCAN cohort. 56% of the i-SCAN group patients had a polyp of any type detected compared to 47% in the standard high definition control group (*p* = 0.03). Adenomatous polyps were detected more frequently in the i-SCAN group compared to the control group (33% versus 27%, *p* = 0.033). On a per patient analysis there were 0.61 adenomas per patient screened in the i-SCAN group versus 0.43 in the control group (*p* < 0.01, Table 2). There were significantly more adenomas detected with i-SCAN in the ascending (*p* = 0.01), transverse (*p* < 0.01), and left colon (*p* = 0.02) with a trend towards increased detection in the cecum (*p* = 0.06).

Significantly more diminutive adenomas were found in the i-SCAN group versus the control group (405 (40.2%) and 260 (28.0%), resp., *p* < 0.01). No difference was found in the i-SCAN versus control group in detecting 6–9 mm adenoma (119 (11.8%) versus 95 (10.2%), *p* = 0.35). More adenomas of 10 mm or greater were found by the i-SCAN group (79 (7.8%) versus 47 (5.1%), *p* = 0.021) (Table 3).

i-SCAN also detected more advanced polyps less than 10 mm based upon histology alone, 37 (3.7%) versus 18 (1.9%) in the control group (*p* = 0.028). i-SCAN did not improve the detection of sessile or flat lesions. 173 sessile/flat adenomas were found in the i-SCAN group compared to 160 in the control group (*p* = 1.00).

## 5. Discussion

Colonoscopy has long been presumed to decrease colorectal cancer by the removal of precancerous adenomatous polyps. Recent publication of long term data from the National Polyp Study has shown that endoscopic removal of adenomas has prevented deaths from colorectal cancer [4]. This finding reinforces the need to maximize detection and removal of adenomatous polyps to prevent colorectal cancer and deaths from it. Several new modalities of enhanced mucosal imaging have been developed with the goal of improving detection of mucosal abnormalities including adenomas. Unfortunately, however, studies of these new technologies have not yet been able to show clear and consistent improvement in adenoma detection rates (ADR).

NBI has been the most widely studied modality with several randomized trials evaluating its ability to improve upon the adenoma detection rate over standard or high definition white light endoscopy (WLE) [7–10]. A recent Cochrane review and meta-analysis found that NBI did not significantly increase adenoma detection rates compared to WLE [11, 12]. In the Cochrane review 8 studies were included involving 3673 patients. No significant difference was found in adenoma detection rate (RR 0.94, CI 0.87–1.02) or total polyps detected (RR 0.97, CI 0.91–1.04) [11]. The meta-analysis included 2936 patients from 7 studies and found no significant difference in adenoma detection rate (*p* = 0.413) or overall polyp detection (*p* = 0.289) [12]. FICE has also been studied and as with NBI it did not show an improved ADR in 2 separate studies involving 1318 patients (ADR 0.28 in both groups) and 359 patients (ADR 0.64 versus 0.55, *p* = 0.65) [13, 14].

Alternatively, studies to evaluate the efficacy of high definition colonoscopy alone compared to standard white light video endoscopy in terms of adenoma detection rate have been mixed. A meta-analysis of five studies (4422 patients) comparing HDWLE versus standard WLE found a pooled weighted mean difference in detection of small adenomas that favored HDWLE (+3.5%, CI 0.9%–6.1%). The authors did caution that the results needed to be interpreted carefully given differences in the designs of the studies included [15].

The ability of i-SCAN to detect colonic neoplasia and predict polyp histology compared to chromoendoscopy was first studied by Hoffman et al. in a group totaling 69 patients. The study showed that i-SCAN is as sensitive as chromoendoscopy in detecting adenomas < 5 mm when evaluating the distal 30 cm of the colon during colonoscopy [16]. A second study by Hoffman et al. involving 100 patients in each arm showed that i-SCAN was able to detect significantly more adenomas of all types, including flat lesions compared to standard definition video colonoscopy [17]. A small pilot study by Chan et al. that included 75 patients evaluated the ability of i-SCAN to aid in correct prediction of polyp histology (adenomatous versus nonadenomatous) in small polyps (<10 mm). It did not find a significant difference in histology prediction between the two [18]. A slightly larger prospective cohort study of 84 patients also failed to show a significantly improved histology prediction using i-SCAN over HDWLE [19].

Hong et al. prospectively compared the ability of i-SCAN to detect adenomas and predict histology compared to HDWLE in a modified back-to-back design including a total of 389 patients. They found that the ADR was not increased by i-SCAN compared to HDWLE but thati-SCAN was able to more accurately predict polyp histology. This study casts some doubt on the ability of i-SCAN to increase ADR. However, their design was suboptimal given that the endoscopist first used i-SCAN upon withdrawal followed by HDWLE and that the same endoscopist performed the repeat examination. Given that i-SCAN is thought to be more sensitive in detecting polyps, using i-SCAN first could introduce operator bias by finding polyps that they may not have as easily seen with HDWLE but did detect given their memory of having just evaluated the same segment with i-SCAN [20].

Testoni et al. published a retrospective study of i-SCAN compared to standard white light endoscopy that showed increased detection rates of adenomas that were “nonprotruding” and <10 mm. A total of 1101 colonoscopies were included in this study, 252 with HD+ i-SCAN and 849 using standard WLE [21].  Table 4 provides a summary of* in vivo* studies of i-SCAN limited to real-time evaluation of colonic pathology [22, 23].

To our knowledge, this study is by far the largest known prospective study of i-SCAN compared to HDWLE in average risk individuals. Overall, it shows that i-SCAN is a promising technology with the ability to detect more adenomas than HDWLE. One limitation of this study is that it was performed at a single ambulatory endoscopy center potentially limiting the broad applicability of the data. However, twelve experienced endoscopists were involved in the study. The comparison cohorts were collected at a separate time, but included participants were from the same patient catchment area. The i-SCAN cohort was younger and more male predominant than the control cohort. In regard to adenoma detection, the effects of the differences between the two groups are likely negated as more adenomas are found in males and in older patients. The withdrawal times were longer in the i-SCAN group but this was likely partly influenced by the endoscopists limited clinical experience with i-SCAN prior to the study. This may also suggest complete colon examinations are longer with virtual chromoendoscopy.

Previous studies have shown that i-SCAN was able to better detect flat or “nonprotruding” lesions and small polyps; however, this study did not show increased detection of flat adenomas [16, 21]. The contradictory findings could be explained by some of our reported polyps only having a qualitative size descriptor of “diminutive” without further clarification of flat/sessile shape. Lack of a formally standardized definition of “flat” polyp could also explain our discordant findings. Our findings do concur with increased detection of “small” adenomas although the defined sizes for “small” in the study by Testoni were <10 mm and ours were <6 mm [21].

i-SCAN in mode 1 detects more overall adenomas compared to HDWLE. The largest difference was found in the detection of diminutive adenomas. This study did not find increased detection of flat/sessile adenomas, but there were differences in design compared to previous studies. It did, however, find an increased number of advanced adenomas by both size and histology. Additional large prospective studies should be undertaken to further define the efficacy of i-SCAN in detecting adenomas based on size and shape.

## Figures and Tables

**Table 1:** Patient demographics and bowel preparation.

	i-SCAN	Control	*p* value
Patients	1007	920	
Age	55 ± 6.2	57 ± 7.5	<0.001
% male	49%	45%	0.082
Good/excellent bowel prep.	74.8%	79.3%	0.02

**Table 2:** Polyp and adenoma detection in the i-SCAN versus control groups.

	i-SCAN	Control	*p* value
Any polyp	56%	47%	0.03
Adenomatous polyps	618	402	<0.01
% patients with adenoma	33%	27%	0.033
Adenomas/patient	0.61	0.43	<0.01

**Table 3:** Adenomatous polyp data.

	i-SCAN	Control	*p* value
Diminutive adenoma	405	260	<0.01
6–9 mm adenoma	119	95	0.35
≥10 mm adenoma	79	47	0.021
Flat/sessile adenoma	173	160	1.00

**Table 4:** *In vivo* studies of i-SCAN in real-time evaluation of colonic pathology.

Author/year/[reference]	*N*	Major findings/conclusions
Hoffman et al., 2010 [16]	69	i-SCAN can detect and identify small adenomatous polyps as well as standard chromoendoscopy in the distal 30 cm of the colon

Hoffman et al., 2010 [17]	220	i-SCAN detects more colorectal neoplasia compared to standard video endoscopy; i-SCAN can accurately predict polyp histology

Lee et al., 2011 [22]	142	i-SCAN and narrow band imaging (NBI) have similar efficacy in predicting histology of diminutive polyps compared to high definition white light colonoscopy (both superior to HDWLE)

Testoni et al., 2012 [21]	1101	Compared to standard white light colonoscopy, i-SCAN detects more polyps, specifically flat and small polyps (<10 mm)

Chan et al., 2012 [18]	75	i-SCAN and HDWLE have similar efficacy in predicting small polyp (<10 mm) histology during colonoscopy

Hong et al., 2012 [20]	389	i-SCAN did not detect more polyps or adenomas compared to HDWLE; i-SCAN in mode 2 better predicted polyp histology than HDWLE

Testoni et al., 2013 [23]	542	Nonexpert endoscopists had a similar detection rate of mucosal lesions compared to expert endoscopists when using i-SCAN; when using standard WLE experts detected more lesions than nonexperts

Basford et al., 2014 [19]	84	A single endoscopist was able to predict histology of small polyps (<10 mm) with high accuracy using both HDWLE and i-SCAN; there was no difference in prediction between i-SCAN and HDWLE (both met ASGE performance thresholds)

Bowman, present	1936	i-SCAN detects more adenomas and advanced polyps compared to high definition white light endoscopy
